# Seroprevalence of anti-*Neospora caninum* antibodies in cows of North-Eastern Algeria

**DOI:** 10.14202/vetworld.2019.765-768

**Published:** 2019-06-08

**Authors:** Kamel Miroud, Amar Benlakehal, Rachid Kaidi

**Affiliations:** 1 Research Laboratory of Epidemio-Surveillance, Production and Reproduction, Health, Cellular Experimentation and Therapy of Domestic and Wild Animals, Department of Veterinary Sciences, University Chadli Bendjedid, BP 73, El-Tarf 36000, Algeria; 2 Department of Applied Biology, Institute of Biology, University Larbi Tebessi, Route de Constantine, Tebessa 12000, Algeria; 3 Veterinary Institute, University Saad Dahleb, Route de Soumaa, BP 270, Blida 9000, Algeria

**Keywords:** cows, enzyme-linked immunosorbent assay, *Neospora caninum*, North-Eastern Algeria, reproduction

## Abstract

**Aim:**

The present cross-sectional study aimed at assessing the seroprevalence of *Neospora caninum* infection both at herd and within herd and at determining risk factors that are associated with its seropositivity.

**Materials and Methods:**

A total of 90 cows distributed over seven herds located in two North-Eastern Algerian provinces were blood sampled in order to be tested for the presence of antibodies against *N. caninum* using a commercially available enzyme-linked immunosorbent assay kit.

**Results:**

The individual seroprevalence of *N. caninum* was found to be 12.22%, and six of the seven herds tested had at least one seropositive cow. The logistic regression model revealed that abortion (odds ratio [OR]=29.15) and parity (OR=7.38) were positively associated with the seropositivity of animals on an individual basis.

**Conclusion:**

The study confirms the existence of *N. caninum* infection in cattle in North-Eastern Algeria. However, a widespread infection rate of 85.71% and its significant statistical association with previous abortion (OR=29.15) need further investigations.

## Introduction

*Neospora caninum* is an obligatory, intracellular, apicomplexan protozoan parasite [1], which is distributed worldwide [2]. It was first recognized in dogs in Norway in 1984 [3] and was identified as a new protozoan for the first time in 1988 [4]. This parasite, considered as the major cause of abortion in cattle, can affect other species also [5,6]. Other reproductive problems such as stillbirth, early embryonic mortality, and embryonic resorption have been related to *N. caninum*. The economic losses are due to a decrease in milk yield in those cows that abort, their culling, and a reduced rate of growth and feed efficiency [7-9]. Dogs [10], coyotes [11], and Australian dingoes [12] are recognized as the definitive hosts for *N. caninum*, whereas cattle and many other species (deer, goats, sheep, horses, and water buffalo) are intermediate hosts [2,6]. The life cycle of *N. caninum* consists of vertical transmission and/or a horizontal transmission [2,13]. Many serological tests, among which an enzyme-linked immunosorbent assay (ELISA) is widely used, detect antibody against *N. caninum* and are commercially available [14,15]. These tests are used in epidemiological surveys to assess *N. caninum* infection prevalence, to examine the relationship between exposure to *N. caninum* and outcomes (abortion, reduced milk yield, and cattle culling), and to determine the individual- and herd-level risk factors for *N. caninum* seropositivity [9,15]. *N. caninum* antibody detection in aborted cows’ serum is only indicative of contact with these protozoa and does not prove that neosporosis is the cause of abortion [9,15].

In Algeria, research studies carried out in order to detect or isolate *N. caninum* are scarce. These are limited to the detection of *N. caninum* in the spleen and liver of naturally infected dogs through the use of polymerase chain reaction (PCR) [16], a serological survey of abortive agents in two cattle farms located in the central part of Algeria [17], a study of the risk factors associated with *N. caninum* infection in the north and northeast of Algeria [18], and a seroprevalence study of *N. caninum* in dairy cattle farms in central-northern Algeria [19] and in eastern Algeria [20].

The objectives of the study were two-fold: To estimate the seroprevalence of *N. caninum* antibodies in cattle and to correlate the results with the following risk factors: History of the most recent abortion recorded, parity, breed, and location, measured on an individual basis in two Algerian provinces.

## Materials and Methods

### Ethical approval

Ethical approval is not required for such type of study. Blood samples were collected as per standard collection technique without any stress or harm to the animals.

### Study population and sampling

A total of 90 adult cows making up seven randomly selected herds located in two Algerian eastern provinces, namely Tebessa and Guelma, were blood sampled. Data related to the reproductive history of the animals (abortion and absence of abortion), breed (native and exotic), and parity (multiparous and primiparous) were collected from breeders and their veterinarians.

### Blood collection

10 ml of blood was collected from the jugular vein of each cow, using vacutainer tubes without anticoagulant. The sera were stored at −20°C at the Regional Veterinary Laboratory (El Khroub, Province of Constantine) until analysis.

### Laboratory analysis

Serum samples (n=90) were tested for anti-*N. caninum* antibodies through a commercial ELISA test “CHEKIT Neospora^®^ (IDEXX Laboratory, Netherlands).” They were tested in duplicates according to the manufacturer’s instructions. The results were read using a photometer at a wavelength of 450 nm. The S_450_ nm of duplicates was averaged, and the values of both the positive control and the samples were corrected by subtracting the S_450_ nm of the negative control as presented below:

Sample: OD_sample_ – OD_negative_

Positive control: OD_positive_ – OD_negative_

The results were expressed as percentages using the following formula:


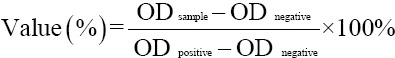


### Statistical analysis

A cow is considered to be seropositive for *N. caninum* if the S/P ratio is ≥30% (manufacturer’s instructions). The seroprevalence rate is the number of cows that are seropositive over the total number of sampled cows, and a herd is defined as seropositive when at least one of its cows test positive to *N. caninum*. Risk factor analysis was performed in two steps: First, the independent variables were subjected to univariate analysis using the Chi-squaretest (n>5) or Fisher’s exact test (n<5) [21] and second, the logistic regression [21] was used to build up a multivariate model to explain the dependent variable (serological status) in relation to the independent variables which had p<0.10 after being subjected to the univariate analysis. The relationship between the serological status and putative risk factors was determined using odds ratio (OR). The statistical significance was determined at p≤0.05. The statistical software package IBM SPSS-USA (version 15.0 for Windows 7, 2006) was used.

## Results

The analysis of the data collected showed that 12.22% of cows and 85.71% of herds were seropositive; the seroprevalences were at the individual level (17.78% and 6.67%) and at a herd level (100% and 33.33%) in Tebessa and Guelma, respectively (Table-1).

**Table-1:** Seroprevalence (%) of *Neospora caninum* infection at individual and herd levels.

Provinces	Herd level			Individual level		
	
	Number of herds	Number of positives	Seroprevalence (%)	Number of cows	Number of positives	Seroprevalence (%)
Tebessa	4	4	100	45	8	17.78
Guelma	3	2	66.67	45	3	6.67
Overall	7	6	85.71	90	11	12.22

### Risk factor analysis

The history of abortion, parity, and breed of cattle were the only three independent variables out of the four investigated that were statistically significantly associated (p<0.10) with seropositivity as shown by the univariate analysis; when these three variables were subjected to multivariate analysis (Table-2), the history of abortion (p=0.000, OR=29.150) and parity (p=0.046, OR: 7.380) proved to be the sole variables significantly associated with seropositivity (Table-3).

**Table-2:** Univariate analysis of the risk factors associated with the seroprevalence of sampled cows.

Factors	Categories	Number tested	Number of positive	% positive	p-value	p Fisher’s exact test
Abortion	Yes	12	5	41.67	0.0007	NA
	No	78	6	7.69		
Parity	Multiparous	47	9	19.15	-	0.04114
	Primiparous	43	2	4.65		
Breed	Native	32	8	25.00	-	0.05566
	Exotic	47	3	6.38		
Province	Tebessa	45	8	17.78	-	0.127
	Guelma	45	3	6.67		

**Table-3:** Result analysis, through the multiple logistic regression, of risk factors associated with the seroprevalence of sampled cows.

Risk factors	Β	SE_β_	p-value	OR	CI 95.0% OR
Breed	1.099	0.888	0.216	3.001	0.527–17.102
*Parity	1.999	1.004	0.046	7.380	1.032–52.797
*Abortion	3.372	0.88	0.000	29.150	5.193–163.623

β=Standard coefficient, SE=Standard error. OR=Odds ratio, CI=Confidence interval,

* p<0.05

## Discussion

The results of data analysis have to be interpreted with care because of the relatively small number of animals that were sampled (90 cows); the latter was mainly due to the refusal of many breeders to take part in the study. The result obtained proved, for the first time, the presence of *N. caninum* in cattle in Tebessa and Guelma. The individual seroprevalence (12.22%) recorded was lower than that reported by previous studies: 19.64% in North-Eastern Algeria [18], 32.8% in central-north Algeria [17], and 16% in Constantine [20] but similar to that reported (12.37%) in a study carried out in five provinces of central-northern Algeria [19]. The discrepancy in the percentage recorded could be due to the serological type test used; cutoff level used to determine the exposure, region, sample size, sampling frame; and livestock farming [2,22], all known to exert a different effect on the results.

The herd seroprevalence rate recorded (85.71%) (Table-1) showed that *N. caninum* infection was highly disseminated across the sampled herds. Although this result is of a low statistical significance due to the limited number of herds sampled. Its clinical importance is, however, greater if one takes into account the high performance of ELISA test (high sensibility and specificity: Se and Sp >95%) [14,15,23], the capacity of its horizontal and vertical transmission [2,13], and the absence of prophylactic measures as it is the case in Algeria.

The cows with the most recent abortion history were more likely to be seropositive (p=0.000, OR=29.15). This is in accordance with what was reported in some studies [18,24-26] where the estimated OR indicated a higher proportion of seropositivity in cattle with than those without an antecedent of reproductive problems. Although *N. caninum* is among the most important abortion-causative agents in cattle worldwide, a significant statistical link between abortion and seropositivity did not fully implicate *N. caninum* as an abortive agent [27]. This can be due to the fact that (a) conclusive data can be obtained only by prospective cohort and experimental studies or direct diagnostic test [2,28,29] and not through cross-sectional studies because they do not take into account the temporal relationship between the risk factors and the outcome [21]; (b) incidence of other abortive agents may mask such an association or decrease the power of analytical techniques to detect it [30]; and (c) ELISA is an indirect diagnostic method in that the detection of antibodies from *N. caninum* bovine serum is only indicative of a contact with these protozoa and does not confirm neosporosis as a cause of abortion, while the negative serological result for *N. caninum* excludes its implication in abortion [31]. To confirm that *N. caninum* is responsible for abortion, other diagnostic techniques such as PCR analysis, histopathologic findings, or immunohistochemical staining are required [7,27,28].

A relationship between the multiparity variable and seropositivity to *N. caninum* (p=0.046, OR=7.38), as reported by previous studies [30,32], was observed in the present work. These authors suggest that the horizontal transmission through oocyst ingestion is particularly important in some herds. This relationship could also be the result of the absence of selective culling of animals seropositive to *N. caninum* as well as that of neosporosis screening in the herds under study [2].

## Conclusion

The present study confirms, for the first time, the existence of *N. caninum* infection in cattle in North-Eastern Algeria. However, the widespread infection rate (85.71%) and its statistically significant association with previous abortion (OR=29.15) require further investigations through more epidemiological studies in order to assess the real infection incidence and to implement surveillance programs to control any eventual progression and transmission of the disease.

## Authors’ Contributions

KM designed and carried out the research study along with AB, who did most of the field work. RK helped with the design and correction of the manuscript. All authors read and approved the final manuscript.
